# Group intervention for non-suicidal self-injury in college students guided by the Process Model of Emotional Regulation: a pilot randomized controlled trial

**DOI:** 10.3389/fpsyt.2026.1863053

**Published:** 2026-09-03

**Authors:** Miao Yu, Shi-Min Chen, Chen-Ying Yu

**Affiliations:** 1 Organization Department of Party Committee, Yancheng Institute of Technology, Yancheng, China; 2 Jianghuai Institute of Humanities, Yancheng Institute of Technology, Yancheng, China; 3 Department of Psychology, Huaiyin Normal University, Huai’an, China; 4 School of Electrical Engineering, Yancheng Institute of Technology, Yancheng, China

**Keywords:** college students, emotion regulation strategies, group intervention, non-suicidal self-injury, Process Model of Emotional Regulation

## Abstract

**Objective:**

Emotion regulation difficulties are a key risk factor for non-suicidal self-injury (NSSI) behaviors. This study aimed to reduce self-injurious behaviors among college students by employing various emotional regulation strategies—including distraction, emotional expression, self-focused and situation-focused cognitive change, and problem-solving—based on the Process Model of Emotional Regulation.

**Methods:**

A total of 56 college students with NSSI behaviors participated in the study, with 28 participants in the experimental group and 28 in the control group. The experimental group received eight sessions of group intervention, while the control group received no intervention. The Self-Rating Anxiety Scale, Beck Depression Inventory-II, and Adolescent Non-Suicidal Self-Injury Assessment Questionnaire were used at three time points: pre-intervention, post-intervention and 2 months’ follow-up. An open-ended questionnaire was used to investigate intervention benefits, and a single-item measure was adopted to assess intervention satisfaction.

**Results:**

This open-ended investigation showed that group members benefited in three aspects: internal changes, external changes, and meaningful experiences. The intervention group demonstrated significant reductions in the levels of anxiety, depression and NSSI behaviors after the intervention, whereas the control group showed no significant changes over the same period. Group members reported high overall satisfaction with the intervention (4.82 ± 0.39).

**Conclusion:**

Guided by the Process Model of Emotion Regulation, this preliminary multi-strategy emotion regulation group intervention is feasible and acceptable, and linked to decreased severity of anxiety, depression, and NSSI among college students.

## 1 Introduction

Non-suicidal self-injury (NSSI), commonly referred to as self-injury, is defined as the direct and intentional destruction of one’s own body tissue without suicidal intent, in a manner not accepted by society or culture (1, 2). Common forms of self-injury include cutting, hitting, burning, scratching, biting, and banging one’s head against hard objects. Adolescents are a high-risk group for self-injurious behavior. A meta-analysis has indicated that the prevalence of self-injury among Chinese middle school students reaches 22.37% (3). In contrast, the prevalence of self-injury among college students shows a declining trend. A meta-analysis has revealed that the prevalence of self-injury among college students is 16.6%, with a slightly lower rate among male students (16.2%) compared to female students (17.8%) (4). Previous studies have indicated that self-injury is significantly associated with various mental disorders, including mood disorders, anxiety disorders, post-traumatic stress disorder, eating disorders, substance abuse, conduct disorder, intermittent explosive disorder, and borderline personality disorder (5, 6). Moreover, and more alarmingly, self-injurious behavior may undergo a dynamic developmental process: initially serving as a short-term coping mechanism, but as behavioral patterns become entrenched, it may form a vicious cycle through negative reinforcement mechanisms, ultimately significantly increasing the risk of suicidal ideation and suicidal behavior (6, 7). Given the multiple risks associated with self-injury, it holds significant practical importance to thoroughly investigate its underlying mechanisms and develop effective intervention strategies.

### 1.1 Emotion regulation difficulties

NSSI behaviors result from the combined effects of multiple risk factors. Beyond environmental risk factors such as insecure attachment (8, 9) and childhood trauma (10, 11), difficulties in emotion regulation constitute a critical intrinsic predictor (12, 13). Self-injury is even regarded as a maladaptive emotion regulation strategy (14, 15). Individuals who engage in self-injury often lack adaptive emotion regulation strategies and overuse maladaptive ones. Previous studies have indicated that individuals with more severe self-injurious behaviors are less likely to employ adaptive emotion regulation strategies including acceptance, cognitive reappraisal, and problem-solving (16–19), while relying more on maladaptive emotion regulation strategies like self-blame, blaming others, catastrophizing, rumination, and expressive suppression (16, 19, 20). Network analyses have identified robust associations between expressive suppression and NSSI behaviors (21, 22). Furthermore, existing evidence indicates that alexithymia contributes to heightened use of maladaptive strategies (e.g., expressive suppression) and reduced engagement in adaptive strategies such as cognitive reappraisal, which in turn elevates the risk of engaging in NSSI (20, 23). In the face of emotional distress, those who engage in self-injury are often deficient in the skills needed to utilize adaptive regulation strategies for managing distress, solving problems, or obtaining support. As a result, they may adopt self-injury as a functionally maladaptive behavior aimed at pain reduction, attention-seeking, or reality avoidance (1, 24).

### 1.2 Intervention for NSSI

Given that emotion regulation difficulties are a key risk factor for self-injurious behavior, assisting individuals who engage in self-injury to adopt a variety of adaptive emotion regulation strategies and enhancing their emotion regulation abilities constitutes an important measure in self-injury intervention. A variety of intervention therapies have been employed, including cognitive behavioral therapy (25), dialectical behavior therapy (26, 27), positive psychological intervention (28), narrative therapy (29), mindfulness intervention (30), art therapy (25, 31), family therapy (32), sports therapy (33), online intervention (26, 34), and imagery therapy (35, 36). The majority of participants in these intervention studies were adolescents recruited from psychiatric hospitals or outpatient psychology clinics (25–32, 34). Meanwhile, several studies also enrolled adolescents recruited from middle schools (33) as well as college students (35, 36). These intervention methods employ various emotion regulation strategies, such as cognitive change, distraction, emotional expression, Family system restructuring, to alleviate negative emotions in individuals who engage in self-injury and to enhance their emotion regulation abilities.

### 1.3 Process model of emotion regulation

**Figure 1:**
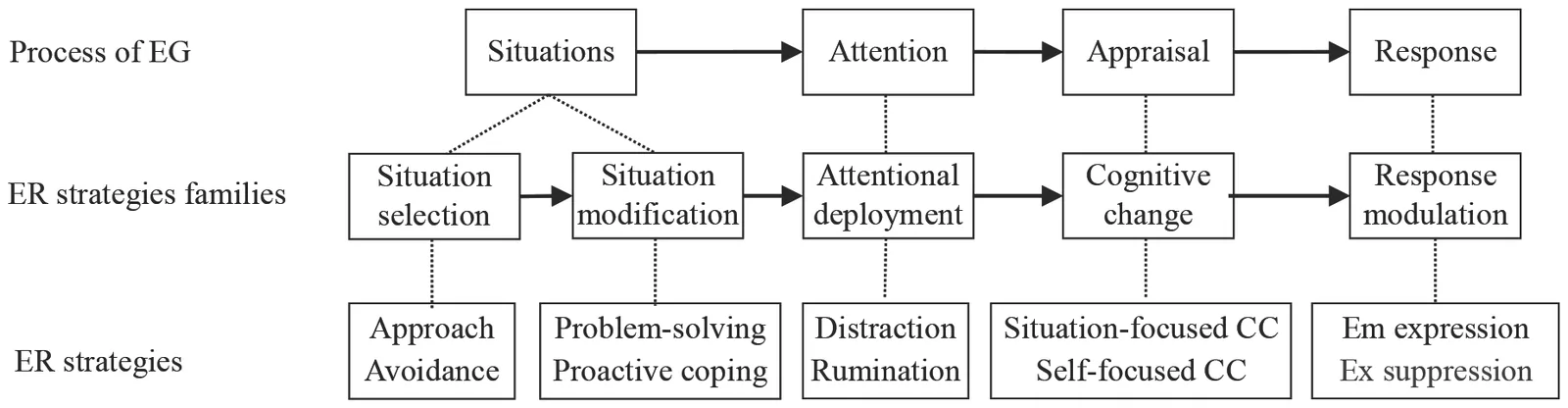
Emotion regulation strategies based on the Process Model of Emotion Regulation. EG, emotion generation; ER, emotion regulation; CC, cognitive change; Em expression, emotional expression; Ex suppression, expressive suppression. Source: Adapted from Gross (37) under license ID 1772838-1.

Previous studies have confirmed the effectiveness of a wide range of interventions for self-injury and emotion regulation strategies. However, most of these interventions only adopt one or a small number of such strategies. How to integrate diverse emotion regulation strategies to improve the emotion regulation ability of individuals with self-injurious behaviors and reduce their risk of self-injury remains a question worthy of further exploration. Grounded in the Process Model of Emotion Regulation (37), this study proposes a self-injury intervention program that systematically incorporates multiple emotion regulation strategies with the aim of enhancing its overall effectiveness. Based on the four stages of the emotional generation process—situation, attention, appraisal, and response—the process model of emotion regulation proposes five families of emotion regulation strategies (**Figure 1**), including situation selection, situation modification, attention deployment, cognitive change, and response modulation (37). Each strategy family involves several strategies (38). When encountering a stressful situation, an individual first decides whether to approach or avoid it. If the individual chooses to approach the situation, they will attempt to modify it. This modification strategy includes both proactive coping before the stressful event occurs and reactive coping (problem-solving) after the event has taken place. When the problem is difficult to resolve, the individual may choose to either continue thinking about it (rumination) or temporarily shift their attention (distraction). According to the Process-specific Timing Framework Theory (39), distraction helps reduce high-intensity negative emotions to a lower intensity. At this point, the individual can then engage in cognitive reappraisal of the lower-intensity negative emotions. Cognitive reappraisal includes two approaches: altering the perception of the situation and changing self-perception (40, 41). Once the emotional experience is fully formed, the individual may choose to either express it or suppress it. Naturally, in real-world contexts, the use of emotion regulation strategies is not a rigidly sequential process but rather a dynamic and flexible one, tailored to the practical situation. Among these strategies, distraction, emotional expression, self-focused cognitive reappraisal, situation-focused cognitive reappraisal, and problem-solving are generally considered adaptive emotion regulation strategies.

Self-injurious behavior is a complex psychological issue, and the integrated application of multiple intervention strategies can yield more optimal outcomes. Therefore, this study aimed to enhance emotion regulation abilities among college students with self-injurious behaviors and reduce their engagement in self-injury by drawing on the theoretical framework of the Process Model of Emotion Regulation and comprehensively adopting a range of strategies, including distraction, emotional expression, self-focused and situation-focused cognitive change, and problem-solving.

## 2 Methods

### 2.1 Participants

*A priori* power analysis was performed using G*Power 3.1.9.7 to calculate the minimum required sample size. The statistical test was set to ANOVA: Repeated measures, within-between interaction. We set α = 0.05, power (1−β) = 0.8, and adopted a medium effect size of f = 0.25. The number of groups was specified as 2, the number of repeated measurements as 3, the correlation among repeated measures as 0.6, and the non-sphericity correction ϵ as 0.75. The results indicated that the minimum sample size per group was 28 participants.

This study aimed to identify 100 valid samples for matching the intervention and control group. According to meta-analytic findings, the prevalence of non-suicidal self-injury among college students is 16.6%. Assuming that one-third of eligible students are willing to participate in the intervention, the required initial survey sample size is calculated as: 100 ÷ 16.6% ÷ (1/3) ≈ 1807 participants.

Participants were screened and recruited according to the following steps. First, ethical approval was obtained from the Academic Ethics Committee at the corresponding author’ affiliated institution. Second, three questionnaires, including the Self-Rating Anxiety Scale (SAS) (42), the Beck Depression Inventory-II (BDI-II) (43), and the Adolescent Non-Suicidal Self-Injury Assessment Questionnaire (ANSSIAQ) (44), were uploaded to the website of “Questionnaire Star”, a widely used online survey platform in China.

**Figure 2:**
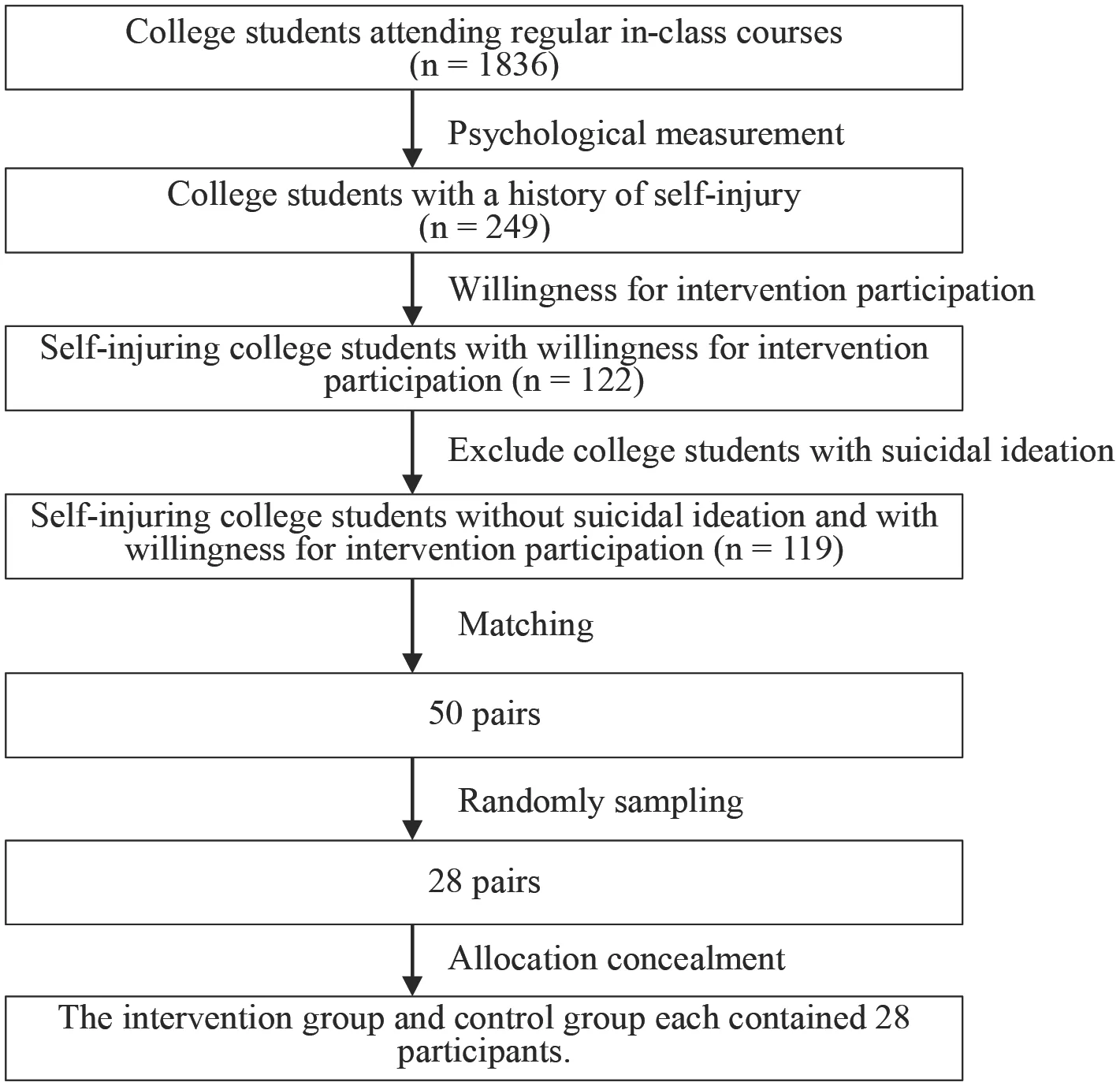
Participant flow diagram.

Third, group administration of the tests was conducted with college students. During a regular class session, the administrator explicitly elaborated on the test’s objectives, confidentiality guidelines, voluntary participation principle, and the total number of test items to the students. Written informed consent was obtained from all participants after a full explanation of the procedure. Subsequently, a QR code linked to the questionnaire was presented on the PPT, enabling students to scan it for questionnaire completion. Upon submission of the filled-out questionnaire, each participating student was provided with a small token of appreciation. A total of 1,836 college students successfully completed the questionnaire survey (**Figure 2**).

Fourth, college students with a history of self-injury behaviors who are willing to participate in this study were screened. According to the survey results, 249 students had engaged in NSSI within the past six months, accounting for 13.6% of the total participants. Among these 249 individuals, 122 (49.0%) expressed willingness to participate in this study.

Fifth, college students with suicidal ideation were excluded. Given the relationship between NSSI behaviors and suicide, suicide risk assessment is essential when working with individuals who engage in self-injury (45, 46). Suicide ideation was screened via Item 9 of the Beck Depression Inventory-II (BDI-II). Three participants endorsed the option “I would like to kill myself”. The three participants were referred to the university counseling center. For safety reasons, they were excluded from the subsequent group intervention.

Sixth, college students with self-injury behaviors were matched. For the remaining 119 college students with self-injury behaviors, a block randomization method was used for group assignment. Participants were first ranked by self-injury scale scores and paired according to score similarity. During matching, other assessment scores were taken into account in addition to self-injury scores. To prioritize matching accuracy, this study only yielded 50 valid matched pairs instead of forcing all 119 college students into pairs. Subsequently, 28 matched pairs were randomly sampled from the full matching dataset.

Seventh, allocation concealment was used to assign the two participants in each pair to the intervention group and the control group. The specific procedures were as follows. (1) The 28 matched pairs were numbered from 1 to 28, and the two participants in each pair were labeled Participant A and Participant B respectively. (2) Twenty-eight opaque sealed envelopes were prepared and numbered 1 to 28. (3) A third-party researcher who was not involved in participant recruitment or intervention generated 28 binary random numbers (0 or 1, each with a 50% probability) via computer, which were placed into the envelopes with matching serial numbers in sequence. (4) When each pair of participants was enrolled, the envelope corresponding to the pair’s serial number was opened. If the random number was 1, Participant A in the pair was assigned to the intervention group and Participant B to the control group; if the random number was 0, Participant A entered the control group and Participant B the intervention group. In the end, 28 participants were included in the intervention group and another 28 in the control group. The intervention group received eight sessions of group intervention (see Section 2.2), whereas participants in the control group received routine campus mental health literacy sessions, including mental health classes, awareness-promoting activities, and themed class meetings.

In addition, this study was designed as a pilot and feasibility randomized controlled trial (RCT), with primary objectives centered on evaluating intervention feasibility, recruitment protocols, and treatment fidelity, rather than establishing causal effectiveness. Notably, given the pragmatic nature of this campus-based trial and its focus on preliminary parameter estimation, prospective trial registration was not pursued prior to enrollment.

The intervention group consisted of 13 males and 15 females, aged 18–21 years (M = 18.64, SD = 0.99), including 16 science and engineering students and 12 liberal arts students, with 16 freshmen, 8 sophomores, 3 juniors, and 1 senior. The control group included 10 males and 18 females, aged 18–22 years (M = 19.64, SD = 1.03), comprising 14 science and engineering students and 14 liberal arts students, with 7 freshmen, 11 sophomores, 7 juniors, and 3 seniors. An independent samples t-test for mean differences revealed no significant difference in self-injury levels between the intervention and control groups (intervention group: M = 5.89, SD = 6.26; control group: M = 6.04, SD = 6.38; *t* = -0.085, *p* > 0.05).

### 2.2 Group intervention program

#### 2.2.1 Facilitators and co-facilitators

Group intervention facilitators were required to hold a psychological counselor certificate with abundant group intervention experience. The two mental health teachers conducting the intervention in this study satisfy the requirements. Prior to the formal delivery of group interventions, the two facilitators conducted multiple rounds of discussions covering the entire intervention process. They standardized the guiding, explanatory and concluding scripts for each session module to minimize implementation bias arising from individual differences in verbal expression, thereby ensuring high consistency in the delivery procedures of all group interventions.

To enhance the group’s heterogeneity, four co-facilitating students in good mental health—with normal levels of anxiety and depression and no history of NSSI—were recruited to serve as peer role models. Prior to intervention, no additional training was provided to the four students. They were merely instructed to take an active part in the activities to improve program effectiveness. All group members, including the four co-facilitators and participants, were kept unaware of one another’s personal information. The participants were not informed of the role of the co-facilitating students. This operational approach has achieved favorable results in our previous group interventions.

#### 2.2.2 Intervention and measurement schedule

Participant recruitment was conducted during Weeks 1 to 3 of the semester, and the intervention group received group intervention from Week 4 to Week 11. Sessions were delivered once weekly, lasting two hours each. To minimize the impact on participants’ daily life, the group intervention was scheduled from 7:00 p.m. to 9:00 p.m. every Friday after consultation with all participants.

The first posttest was administered within three days upon completion of the intervention. Existing intervention studies adopt different timelines for the initial posttest assessment: some conduct measurements immediately after the final intervention session, while others implement assessments 1 to 3 days post-intervention. Compared with immediate post-session testing, a 1–3 day delay can mitigate social desirability bias to a certain extent and avoid overestimating intervention effects driven by transient elevated positive affect right after activities. Therefore, the first posttest was arranged within three days following the end of all intervention sessions. A follow-up assessment was administered two months later, after all participants had finished their semester examinations.

#### 2.2.3 Intervention setting and materials

This intervention was conducted in the school group counseling room. Equipped with a projector, the room supported lectures on theoretical knowledge relevant to group intervention. All wheeled desks could be moved flexibly to rearrange the space and meet the demands of group activities.

Materials adopted for the core activities of this intervention include A3 and A4 printing paper, scissors, adhesive tape, paintbrushes, and Holland Career Planning Cards. To accommodate the testing requirements of group intervention, the Holland Occupational Interest Test contained therein was entered into a computer and printed uniformly.

#### 2.2.4 Intervention sessions

Eight face-to-face group intervention sessions were arranged. Each intervention session consisted of three segments: a warm-up activity, core activities, and a sharing session. The time allocation is specified as follows: 5–10 min for warm-up, 20–25 min for sharing; the core part includes 2 to 3 sub-activities with a total duration of 90 min. The objective, adopted emotion regulation strategies, core activities, schedule and homework of each intervention are presented in **Table 1**.

**Table 1:** Core activities for group intervention on NSSI among college students.

NO	Sessions	Objective	Core activities & time allocation	Homework	ER strategies
1	Team building	(1) Enable members to get acquainted with one another. (2) Foster a harmonious and supportive group atmosphere. (3) Clarify the ground rules of the group.	(1) Grouping, group name creation, group leader selection, name-based storytelling, 40min. (2) Collaborative drawing, 40 min. (3) Group agreement clarification & Signature, 10 min.		
2	Self-exploration	Assist members in discovering more personal strengths and boost their self-confidence	(1) Select an animal to represent yourself and analyze your strengths,20 min. (2) Tree drawing test and analyze your strengths, 35 min. (3) Bombardment of strengths, 35 min	Homework 1	SeFCC
3	Relaxation training	Help members learn relaxation skills and reduce negative emotions.	(1) Breathing relaxation, 5 min. (2) Muscle relaxation, 5 min. (3) Mindfulness meditation, 10 min. (4) Play your favorite song and explain why you like it, 70 min.	Homework 2	Distraction EE
4	Family relationship	(1) Enable members to express their feelings towards their families. (2) Help participants recognize the warmth within their families.	(1) Family drawing Test, 45 min. (2) Describe the situations in your home that makes you feel warm, 45 min.		EE SiFCC
5	Conflict resolution	(1) Help members become aware of their own conflict management styles. (2) Guide participants to learn to resolve conflicts through negotiation.	(1) Perform an interpersonal conflict psychodrama and reflect on the style you use when facing conflicts, 45 min. (2) Rewrite the psychodrama in a negotiated way, 45 min.		EE PS
6	Goals & plans	(1) Explore personal vocational interests (2) Establish future goals and plan	(1) My role model: Who is your role model? What are his/her strengths? What do you intend to learn from him/her? 30 min. (2) Explore vocational interests via Holland’s career planning cards, 30 min. (3) Draw titled “Past, present, and future”, and establish your goals and plans, 30 min.		PS
7	Goal achievement: Strengths & Ways	Identify available resources and strategies to achieve personal goals	(1) My success story: What is your success story? What challenges did you face? How did you overcome it? What strengths did this experience reveal about you? 45 min. (2) Share learning experiences and methods, 45 min.	Homework 3	SeFCC PS
8	Heartfelt farewell	(1) Recognize positive aspects of life and cultivate gratitude toward others. (2)Hold a closing ceremony for the whole group counseling program	(1) Share: benefits and personal growth from this group program, 50 min. (2) Reciprocal gratitude expressions among members (Gift presentation optional), 30 min. (3) Farewell ceremony, 30 min.		SeFCC

Homework 1: (1) Select your favorite song; (2) Explain why you like it; (3) Forward the MP3 file to the group facilitator. Homework 2: Jog at least 3 times per week, for a minimum of 15 minutes each time. Complete check-in in the WeChat group after finishing running. Homework 3: (1) Who would you like to express gratitude to during this program? (2) How would you like to convey your appreciation? (Gift presentation optional) ER Strategies. emotion regulation strategies, EE. emotional expression, SeFCC. Self-focused cognitive change, SiFCC. Situation-focused cognitive change, PS. problem solving.

During the first session “Team Building”, participants and co-facilitators were briefed on and asked to sign a group agreement. The agreement includes the following provisions: (1) Voluntary Participation. Group members may withdraw from the group activities at any time with prior notification. Once they drop out, they will not be allowed to rejoin. In addition, all sharing within the group is voluntary: participants may choose freely whether to share or not, and may stop speaking at any point if they feel uncomfortable during sharing. (2) Principle of respect. This principle entails respecting every member’s willingness to share, as well as their right to remain silent. It calls for embracing diverse emotions and individual differences, refraining from judgment or belittlement, and avoiding any intrusion on others’ psychological or physical boundaries. Ostracism and ridicule are not tolerated, nor is forcing one’s personal viewpoints onto others. (3) Confidentiality. All content discussed during group activities and members’ personal information must be kept strictly confidential and shall not be disclosed to any external parties. (4) Punctual Attendance. Members are expected to attend group activities punctually at the scheduled time. In case of special circumstances, a leave request must be submitted in advance. (5) Seek help. During group counseling, if you encounter any uncomfortable situation that cannot be properly resolved on the spot, or if an emergency arises after the group session, you may contact the group facilitator for timely support. Group intervention carries multiple potential risks, mainly including interpersonal harm caused by privacy disclosure, emotional deterioration resulting from trauma reactivation, and excessive intrusion into personal boundaries. The principles of voluntariness, respect, confidentiality and help-seeking stipulated in the group agreement can mitigate or prevent the occurrence of the above potential risks to a certain extent.

For Sessions 2 to 8, core activities were designed in accordance with various emotion regulation strategies. First, drawing on self-focused cognitive change strategies, sessions incorporated activities such as “An animal that represents me,” the Tree Drawing Test, “Bombardment of strengths” (Session 2), “My role model,” the Holland Occupational Interest Assessment (Session 6), and “My success story” (Session 7). These activities were designed to help participants develop a comprehensive understanding of themselves, identify their strengths, and clarify their personal interests.

Second, distraction-based strategies were implemented through activities including breathing relaxation, muscle relaxation, mindfulness meditation, and jogging at least three times per week (Session 3). The goal of these exercises was to assist participants in actively redirecting their attention when experiencing intense negative emotions, thereby promoting physical and mental relaxation and facilitating a quicker reduction of such emotions.

Third, emotional expression strategies were integrated into activities such as “My favorite music” (Session 3), the Family Drawing task (Session 4), and Psychodrama focused on interpersonal conflict resolution (Session 5). These tasks encouraged participants to express their innermost feelings, share emotional experiences related to family, and explore emotional responses in interpersonal conflict situations.

Fourth, situation-focused cognitive change strategies informed the design of activities like “Identifying warm family scenes” (Session 4) and “Gratitude expression” (Session 8). These aimed to help participants reframe their perceptions of everyday life, guiding them to actively recognize and appreciate positive aspects of their experiences.

Fifth, grounded in problem-solving strategies and targeting common psychological issues among university students—such as interpersonal conflicts, uncertainties in career planning, and academic challenges—activities were introduced including adapted psychodrama through negotiation (Session 5), drawing “Past • Present • Future,” establishing goals and developing future plans (Session 6), and sharing learning experiences and methods (Session 7). These were intended to enhance participants’ ability to manage interpersonal conflicts, formulate clearer future plans, and improve their learning strategies.

Three homework assignments are arranged throughout the intervention. For the first assignment, participants select a favorite song and submit its audio file to the group facilitator. Two days prior to the third intervention session, the facilitator reminds members who fail to submit the audio to hand in their materials. The second assignment requires participants to go jogging at least three times per week. Participants are encouraged to exercise in pairs and complete check-ins via the WeChat group to foster an atmosphere of healthy competition and mutual supervision among peers. The third assignment asks participants to identify people they feel grateful for during the intervention and formulate corresponding ways to express gratitude. The facilitator sends a reminder via the WeChat group three days before the eighth intervention session.

During the intervention, especially in Section “Self-exploration”, “Family relationship” and “Conflict resolution”, the group facilitators conducted in-depth inquiries into participants’ personal conditions, including their prior suicide attempts, current suicidal ideation, severe depression, psychosis, acute crisis, adverse events, and other relevant clinical risk conditions.

In addition, throughout the intervention and follow-up periods, participants in both the intervention and control groups were required to proactively inform research facilitators if they received any additional counseling or interventions. Participants were instructed to contact research team leaders, class teachers, or the school psychological counseling center upon the occurrence of any adverse events.

#### 2.2.5 Intervention implementation

##### 2.2.5.1 Intervention fidelity

Intervention fidelity is a critical factor ensuring the standardized implementation of interventions. Since the intervention program of this study was delivered by the researcher, the implementer possessed comprehensive mastery of intervention procedures, core techniques and delivery criteria, which helped reduce implementation drift and improve consistency in intervention delivery. Prior to the formal group intervention sessions, the two facilitators conducted multiple rounds of discussions covering the full intervention workflow. They standardized the guiding remarks, explanations and closing summaries for each session component to minimize implementation bias arising from individual differences in verbal expression. This ensured high homogeneity across all group intervention sessions and maintained strong intervention fidelity.

Two approaches were adopted to assess intervention fidelity. First, group facilitators self-rated implementation fidelity on a single-item 5-point Likert scale within the implementation log after each session. Second, all intervention sessions were video-recorded. Two of the recordings (25% sessions) were randomly selected and independently coded by two blind raters unaware of the study hypotheses and group allocation. The raters scored each recording on a 5-point Likert scale in accordance with the fidelity checklist.

Intraclass correlation coefficient (ICC) was used as the indicator to evaluate inter-rater reliability for fidelity assessment. The reliability benchmarks were defined as follows: ICC < 0.40 indicates poor inter-rater reliability; 0.40 ≤ ICC < 0.59 indicates fair inter-rater reliability; 0.60 ≤ ICC < 0.74 indicates good inter-rater reliability; ICC ≥ 0.75 indicates excellent inter-rater reliability.

##### 2.2.5.2 Participant adherence

To rigorously ensure the full implementation of the research intervention, mitigate adherence-related issues such as participant attrition, absence, and perfunctory participation, and maximize the integrity and validity of intervention data, the following strategies were adopted in this study. First, participants were provided with comprehensive information regarding the group intervention, including its purpose, theoretical basis, curriculum modules, schedule, and attendance requirements. Second, engaging thematic activities were designed. Immersive scenario-based experiences were combined with group interaction, reflection sharing and peer feedback to balance activity enjoyment, enrich participant experience and enhance intervention effectiveness. Third, a rigorous attendance and follow-up management system was established. Reminders were sent to all participants via a unified communication channel one day prior to each group session, and formal sign-in records were completed on the day of intervention. For participants who requested temporary leave or missed sessions, the group facilitator conducted gentle one-on-one follow-ups after class to identify reasons for absence. Core intervention content and supplementary task materials were provided individually to these participants, ensuring full exposure to intervention content and preventing disrupted intervention exposure and subsequent declines in adherence caused by single-session absence.

### 2.3 Measures

Three waves of surveys were conducted in this study. The questionnaires in the first wave included the Self-Rating Anxiety Scale (SAS), the Beck Depression Inventory-II (BDI-II) and the Adolescent Non-Suicidal Self-Injury Assessment Questionnaire (ANSSIAQ). The Chinese version of SAS was revised by Wang and Chi (1984) (47). This scale consists of 20 items. Respondents were asked to describe the frequency of various anxiety symptoms, ranging from “none or little (1 point)” to “often (4 points).” The standard score is calculated by multiplying the raw total score by 1.25 and rounding to the nearest whole number. A score between 50–59 indicates mild anxiety, 60–69 indicates moderate anxiety, and 70 or above indicates severe anxiety. The internal consistency reliability of this scale, as measured by Cronbach’s alpha coefficient, was 0.885.

The Chinese version of BDI-II was adapted by Wang et al. (2011) (48). This inventory consists of 21 items. Respondents rate the severity of depressive symptoms using a 0–3 point scale for each item. The total score is the sum of the ratings from all 21 items. A score between 14–19 indicates mild depression, 20–28 indicates moderate depression, and 29 or above indicates severe depression. The internal consistency reliability of the scale was Cronbach’s α = 0.916.

The 12-item ANSSIAQ was developed by Wan et al. (44), and validated with Chinese college students by Yin et al. (49). It comprises two dimensions: (1) self-injurious behaviors without significant tissue damage (e.g., intentionally pinching oneself or punching walls), and (2) self-injurious behaviors with significant tissue damage (e.g., deliberately cutting or burning oneself). Respondents are asked to report the frequency of their self-injurious behaviors over the past two months, with options ranging from “never (0 points)” to “often (4 points)”. Higher total scores indicate more severe self-injurious behaviors. The internal consistency reliability of the questionnaire, as measured by Cronbach’s alpha coefficient, was 0.918.

The second wave of survey was administered within three days post-intervention, using the SAS, BDI-II, and ANSSIAQ questionnaires with both the intervention group and the control group. Two additional items were included for the intervention group. The first item was an open-ended question asking participants to describe what they benefited from the group intervention. The second item rated the intervention satisfaction on a 5-point scale, ranging from “very dissatisfied (1 point)” to “very satisfied (5 points)”.

The third survey was conducted two months after the intervention, utilizing three questionnaires: the SAS, BDI-II, and ANSSIAQ.

All questionnaires were distributed via “Questionnaire Star” online survey platform. The platform’s forced response function was enabled, ensuring complete responses to all questionnaire items. In addition, for the second and third waves of surveys, facilitators sent WeChat reminders to participants who failed to complete the questionnaires within the designated time frame. Collectively, these measures effectively prevented missing data.

### 2.4 Data analysis

The analysis of intervention benefits was conducted based on the three-level coding principles of grounded theory, utilizing the qualitative analysis software of NVivo 11. The procedure was as follows. First, during the open coding stage, line-by-line analysis of raw textual data was performed to identify and extract key concepts, forming free nodes (the third-level nodes). If a single participant provides multiple phrasing variations for the same benefit content, only one coding entry is counted. Subsequently, in the axial coding stage, through constant comparison, logical relationships among the third-level nodes were systematically analyzed, and intrinsically related concepts were categorized and integrated to form tree nodes (the second-level nodes). Finally, during the selective coding stage, the researcher employed theoretical sampling and continuous comparison to further refine core relationships among secondary nodes, ultimately deriving highly generalized case nodes (the first-level nodes) and establishing a comprehensive conceptual framework of intervention benefits. Two researchers independently coded the data according to the aforementioned procedure, followed by a joint review of discrepant codes. Percentage agreement was employed to evaluate coding consistency, calculated as number of mutually agreed codes/(number of mutually agreed codes + number of mutually disagreed codes) × 100%. The two researchers achieved an initial percentage agreement of 81.4%, which rose to 96.2% following joint review. For codes remaining unresolved after review, a third researcher was invited to arbitrate and render a final ruling.

The intervention integrated solid theoretical foundations with engaging activities, which was well-received by all participants. High participant adherence was observed with zero attrition throughout the trial. This study adopted the intention-to-treat (ITT) analysis principle. Since all randomized participants completed the full intervention without dropouts, complete datasets from all initially allocated cases were included in analyses, with grouping determined by the original random assignment.

Data were processed using SPSS 26.0. A mixed-design ANOVA was conducted to compare changes in anxiety, depression, and NSSI across groups. The analysis included a between-subjects factor (group: intervention vs. control) and a within-subjects factor (time: pre-intervention, post-intervention, follow-up). The following steps were adopted to perform a mixed-design ANOVA. First, Mauchly’s test of sphericity is conducted for within-subject factors with three or more levels. When *p* > 0.05, the sphericity assumption is met, and the uncorrected F-value should be used; when *p* < 0.05, the sphericity assumption is violated, and correction is required. The correction method is as below: if Epsilon ≥ 0.75, apply the Huynh–Feldt correction; if Epsilon < 0.75, apply the Greenhouse–Geisser correction. The corrected degrees of freedom are obtained by multiplying the uncorrected degrees of freedom by the Epsilon value. Second, between-subjects main effects, within-subjects main effects, and interaction effects are tested. Third, if the interaction effect is significant, simple effects tests are performed with a syntax program using the EMMEANS command. Partial eta-squared (*η*^2^) was used as the effect size measure for ANOVA. According to established criteria, effect sizes were interpreted as follows: partial *η*^2^ < 0.059 indicates a small effect size; 0.059 ≤ partial *η*^2^ < 0.14 indicates a medium effect size; and partial *η*^2^ ≥ 0.14 indicates a large effect size. In addition, considering that year of study may affect anxiety levels, it was included as a covariate in the mixed-design ANOVA for anxiety.

## 3 Results

### 3.1 Implementation of the intervention

The intraclass correlation coefficient (ICC) for treatment fidelity scale ratings by two independent coders was 0.821, indicating excellent interrater reliability. The mean self-reported implementation fidelity score of intervention facilitators was 4.63, while the mean fidelity score rated by coders was 4.56, demonstrating satisfactory implementation fidelity of the group intervention.

The overall implementation of this group intervention yielded favorable outcomes. Participants demonstrated high cooperation throughout the study. No participants dropped out midway or were absent without cause. All enrolled subjects completed the full intervention sessions, conscientiously finishing interactive tasks at each stage and post-intervention surveys. The overall participant adherence was satisfactory, which effectively guaranteed the integrity of the intervention and the reliability of the study findings.

Furthermore, throughout the intervention and follow-up period, none of the participants accessed additional counseling or external intervention services, and no reports of severe adverse events were documented.

### 3.2 Intervention benefits

All 28 participants in the intervention group completed the open-ended survey. Their perceived benefits from the self-injury intervention are presented in **Table 2**. Frequencies listed in the table correspond to coded entries. Since multiple descriptions of the same benefit reported by a single participant are consolidated into one coding count, frequencies match the number of participants on a one-to-one basis. Through open coding, 28 third-level nodes were identified. During the process of refining third-level nodes into second-level nodes, the internal relationships among the third-level nodes as well as the relevant emotion regulation strategies were taken into consideration. Through categorizing and integrating these third-level nodes, a total of 9 second-level nodes were ultimately derived.

**Table 2:** Benefits from this self-injury intervention.

1st-level nodes	2nd-level nodes	3rd-level nodes(Frequency)	Related ER strategies
Internal changes	Self-change	Gaining a better understanding of myself (16)	All strategies, esp. self-focused cognitive change
		Gaining a better understanding of my family(4)	
		Opening up my heart (4)	
		Becoming more confident (5)	
		Learn to motivate myself (2)	
		Becoming more composed (4)	
	Improving emotion regulation ability	Learning to relax myself (8)	Distraction
		Becoming more positive and optimistic (4)	Cognitive change
		No longer dwell on the past (2)	
		Believing that there are kind people in this world (2)	
	Changes in learning	Learning more psychological knowledge (3)	Problem-solving
		Improving learning methods(4)	
		Learning from others’ experiences (7)	
	Improving interpersonal skills	Improving interpersonal skills (10)	
	Clearer goals	Future goals have become more explicit (8)	
	Addressing psychological distress	Resolving emotional knots(4)	Emotion expression Cognitive change
	Emotional changes	Being relaxed (23)	All strategies
		Being happy and joyful (20)	
		Being more outgoing(2)	
		Feeling warm (2)	
		Smile a lot more (3)	
		Pressure lessened (5)	
		gloom in my heart dissipated (4)	
External changes	Changes in interpersonal relationship	Gaining recognition from others (2)	Problem-solving
		Improved interpersonal relationships (4)	
Meaningful experience	Meaningful experience	Meeting good teachers and helpful friends (24)	All strategies
		Receiving care and help (8)	
		Unforgettable memories (5)	

ER strategies, emotion regulation strategies.

The seven secondary nodes—self-change, improving emotion regulation ability, changes in learning, improving interpersonal skills, clearer goals, addressing psychological distress, and emotional changes—all pertain to psychological changes in the individual across dimensions such as self-concept, cognition, emotion, and competence. Therefore, they were consolidated into the first-level node of “Internal change.” The node “changes in interpersonal relationships” primarily reflects external transformations at the level of social interaction, and thus was refined into the first-level node of “External change.” Furthermore, participation in the group intervention, as a meaningful experience, served as the precursor for participants’ internal and external changes, leading to the establishment of “Meaningful experience” as another first-level node. Therefore, three first-level nodes—internal changes, external changes, and meaningful experiences—were formed.

### 3.3 Changes in mental health

#### 3.3.1 Changes in anxiety

A mixed-design ANOVA was conducted on anxiety. Mauchly’s test of sphericity was significant (*p* < 0.01), indicating that the sphericity assumption was violated. Since Epsilon = 0.918 > 0.75, the Huynh-Feldt correction was applied. The between-subjects main effect of group was significant, *F*(1, 53) = 4.427, *p* = 0.004. After Huynh-Feldt correction, the within-subjects main effect of time was significant, *F*(1.836, 97.301) = 24.638, *p* < 0.001. The group × time interaction was significant, *F*(1.836, 97.301) = 38.896, *p* < 0.001. Simple effect tests (**Tables 3**, **4**) indicated that anxiety levels decreased continuously and significantly in the intervention group, while no significant change was observed in the control group. Prior to the intervention, anxiety levels were significantly higher in the intervention group than in the control group.

**Table 3:** Result of simple effects analysis on changes in the levels of anxiety.

Group	Pre-test		Post-test		Follow-up		*F*	*Post hoc*	Partial *η*^2^
	M	SD	M	SD	M	SD			
Intervention	61.12	3.32	55.27	3.81	53.62	4.82	33.699***	pre>po>fo	0.564
Control	54.78	4.10	55.04	5.13	54.20	6.21	2.369	(pre,po,fo)	0.084
*Post hoc*	In>Co		(In, Co)		(In, Co)				

In, intervention; Co, control; pre, pre-test; po, post-test; fo, follow-up test, ***p < 0.001, > indicates significantly greater than, () indicates that the levels in the parentheses insignificantly differ.

**Table 4:** Simple effect test for within-subject anxiety across fixed groups.

Group	Level 1	Level 2	*p*	CI
Intervention	t1	t2	<0.001	[4.676, 7.021]
	t1	t3	<0.001	[5.806, 9.194]
	t2	t3	0.014	[0.286, 3.018]
Control	t1	t2	1.000	[-1.424, 0.888]
	t1	t3	1.000	[-0.990, 2.151]
	t2	t3	0.287	[-0.407, 2.103]

CI, confidence interval; t1, pre-intervention; t2, post-intervention; t3, follow-up.

#### 3.3.2 Changes in depression

A mixed-design ANOVA was performed on depression. Mauchly’s test of sphericity was significant (*p* < 0.001), indicating a violation of the sphericity assumption. Given that Epsilon = 0.704 < 0.75, the Greenhouse-Geisser correction was applied. The between-subjects main effect of group was non-significant, *F*(1, 54) = 3.818, *p* = 0.056. After Greenhouse-Geisser correction, the within-subjects main effect of depression was significant, *F*(1.408, 76.018) = 42.564, *p* < 0.001. The interaction between group and time was also significant, *F*(1.408, 76.018) = 34.379, *p* < 0.001. Simple effect tests (**Tables 5**, **6**) showed that depressive symptoms decreased significantly after intervention in the intervention group, while no significant change was found in the control group.

**Table 5:** Result of simple effects analysis on changes in the levels of depression.

Group	Pre-test		Post-test		Follow-up		*F*	*Post hoc*	Partial *η*^2^
	M	SD	M	SD	M	SD			
Intervention	18.29	3.40	13.93	3.89	13.21	4.78	28.554***	pre>(po,fo)	0.523
Control	17.68	5.30	17.86	5.37	17.14	5.77	0.853	(pre,po,fo)	0.032
*Post hoc*	(In, Co)		In<Co		In<Co				

In, intervention; Co, control; pre, pre-test; po, post-test; fo, follow-up test; ***p < 0.001, > indicates significantly greater than, () indicates that the levels in the parentheses insignificantly differ.

**Table 6:** Simple effect test for within-subject depression across fixed groups.

Group	Level 1	Level 2	*p*	CI
Intervention	t1	t2	<0.001	[3.544, 5.171]
	t1	t3	<0.001	[3.532, 6.611]
	t2	t3	0.222	[-0.267, 1.696]
Control	t1	t2	1.000	[-1.123, 0.765]
	t1	t3	0.964	[-0.821, 1.892]
	t2	t3	0.244	[-0.294, 1.722]

CI, confidence interval; t1, pre-intervention; t2, post-intervention; t3, follow-up.

#### 3.3.3 Changes in NSSI

A mixed-design ANOVA was conducted on NSSI. Mauchly’s test of sphericity was significant (p < 0.01), suggesting a violation of the sphericity assumption. Since Epsilon = 0.884 > 0.75, the Huynh-Feldt correction was adopted. The between-subjects main effect of group was non-significant, *F*(1, 54) = 2.572, *p* = 0.115. After Huynh-Feldt correction, the within-subjects main effect of self-injurious behavior reached significance, *F*(1.768, 95.485) = 20.426, *p* < 0.001. The interaction between group and time was also significant, *F*(1.768, 95.485) = 21.082, *p* < 0.001. As shown in simple effect tests (**Tables 7**, **8**), the level of self-injurious behavior decreased significantly in the intervention group following the intervention, whereas no significant change was observed in the control group.

**Table 7:** Result of simple effects analysis on changes in the levels of NSSI.

Group	Pre-test		Post-test		Follow-up		*F*	*Post hoc*	Partial *η*^2^
	M	SD	M	SD	M	SD			
Intervention	5.89	6.26	2.79	3.60	2.39	3.62	28.165***	pre>(po,fo)	0.515
Control	6.04	6.38	6.36	6.62	5.86	6.87	1.227	(pre,po,fo)	0.044
*Post hoc*	(In, Co)		In<Co		In<Co				

In, intervention; Co, control; pre, pre-test; po, post-test; fo, follow-up test; ***p < 0.001, > indicates significantly greater than, () indicates that the levels in the parentheses insignificantly differ.

**Table 8:** Simple effect test for within-subject NSSI behaviors across fixed groups.

Group	Level 1	Level 2	*p*	CI
Intervention	t1	t2	<0.001	[2.016, 4.198]
	t1	t3	<0.001	[2.292, 4.708]
	t2	t3	0.705	[-0.415, 1.201]
Control	t1	t2	1.000	[-1.412, 0.770]
	t1	t3	1.000	[-1.029, 1.386]
	t2	t3	0.396	[-0.308, 1.308]

CI, confidence interval; t1, pre-intervention; t2, post-intervention; t3, follow-up.

### 3.4 Intervention satisfaction

Among the 28 participants, 23 reported being “very satisfied” (5 points) with the intervention, while 5 indicated being “relatively satisfied” (4 points). The mean satisfaction score was 4.82, with a standard deviation of 0.39.

## 4 Discussions

### 4.1 Intervention based on Process Model of Emotion Regulation

#### 4.1.1 Intervention and control groups

A between-group controlled design was adopted in this study, with participants allocated to an intervention group and a control group. As illustrated in **Tables 3**, **5** and **7**, no statistically significant between-group differences were found in the levels of depression and NSSI behaviors, whereas participants in the intervention group exhibited a higher level of anxiety than those in the control group. Such discrepancy in anxiety may be attributable to the unbalanced grade composition across the two groups: the intervention group included 16 freshmen, compared with merely 7 in the control group. Previous studies have indicated that first-year undergraduates suffer from adaptation challenges in daily life, academics and interpersonal interactions, leading to significantly higher anxiety than sophomores and juniors (50, 51). Specifically, in terms of daily life, most freshmen relocate to unfamiliar cities for higher education and have to adjust to new surroundings involving dietary habits, accommodation and local climate. Academically, approaches to university study differ markedly from those employed in senior high school. Socially, freshmen break away from their original family and friend networks and need to establish brand-new peer relationships from scratch. The cumulative effect of multiple adaptive stressors contributes to elevated anxiety among freshmen, which further accounts for the higher baseline anxiety indicators observed in the intervention group relative to the control group.

#### 4.1.2 Intervention strategies and effects

The objective of this study was to reduce self-injurious behaviors among college students by enhancing their emotion regulation abilities, based on the Process Model of Emotion Regulation. The intervention designed multiple sessions to regulate participants’ emotions and improve their emotion regulation abilities through strategies such as distraction, emotional expression, self-focused and situation-focused cognitive change, and problem-solving.

First, activities such as selecting representative animals, tree drawing tests, strength bombardment, music appreciation, and stories of overcoming challenges were implemented to assist participants in better understanding themselves, discover more of their strengths, and regulate emotions through self-focused cognitive change strategies. As revealed by the open-ended survey, self-change emerged as a significant intervention benefit for the participants. They “gained a clearer understanding of their own strengths”, “became more self-confident”, “learned to motivate themselves”, and “grew more composed” (**Table 2**).

Second, participants were guided to relax through four distraction-based strategies: breathing relaxation, muscle relaxation, mindfulness meditation, and jogging. These approaches helped the members to shift their attention away from stressful events to enjoyable activities. Open-ended survey indicated that members reported “learning to relax” as a result of the intervention (**Table 2**).

Third, through Family Drawing and interpersonal conflict psychodrama, members gained insight into their family dynamics and conflict resolution styles, while expressing emotions arising from social interactions using emotional expression strategies. Participants reported “resolving emotional knots” and “the gloom in my heart has dissipated” (**Table 2**).

Fourth, by encouraging members to identify warm scenes within their families and express gratitude, they were supported in reshaping perceptions of home and discovering positivity in life, applying situation-focused cognitive change strategies for emotion regulation. After intervention, members became more positive and optimistic, stating they “no longer dwell on the past” and “believe that there are kind people in this world” (**Table 2**).

Fifth, interpersonal conflicts, career planning, and academic issues are the most common problems among college students. Psychodrama rewriting was adopted to help participants enhance their conflict resolution skills; role modeling, Holland’s vocational interest analysis, and the “Past, Present, and Future” drawing activity were used to assist participants in setting goals and formulating plans; and learning method sharing sessions were conducted to improve participants’ learning approaches. These problem-solving strategies helped the group members “enhance interpersonal skills”, “improve interpersonal relationships”, “gain recognition from others”, “refine learning methods”, and “establish more explicit future goals” (**Table 2**).

The integrated application of these strategies yielded positive intervention outcomes. The open-ended survey indicated that the intervention successfully resolved participants’ underlying concerns, enhanced their ability to self-relax, and promoted more positive self-perceptions. Furthermore, it improved their emotional regulation and social skills, alongside significant improvements in learning methods and interpersonal relationships. These positive changes collectively contributed to an increase in positive emotions and a reduction in negative emotions among participants. As shown in **Table 2**, over half of the members reported feeling “relaxed,” “happy,” or “joyful” due to the intervention. Additionally, participants described themselves as “more outgoing,” “feeling warmer,” “smiling more often,” and “less stressed”. This experience was deeply meaningful to them, becoming an “unforgettable memory” (**Table 2**). Scale assessment results revealed significant reductions in participants’ anxiety, depression, and self-injurious behaviors after the intervention. A two-month follow-up found no rebound in these indicators, which indicates the intervention has promising long-term efficacy. Due to the favorable intervention effects, all members have shown a high level of satisfaction with the intervention. Furthermore, the intervention group demonstrated significant reductions in anxiety, depression, and self-injury levels, whereas no significant changes were observed in the control group. These findings indicated that the pilot intervention program based on the Process Model of Emotion Regulation may be feasible and acceptable, and associated with reductions in anxiety, depression, and NSSI behaviors.

#### 4.1.3 Potential effects

When evaluating the intervention effects of this study, other potential confounding effects should also be taken into account, including the self-selection effect, regression effect, natural recovery effect, baseline differences, social desirability effect and possible influences from co-facilitators.

The self-selection effect refers to the phenomenon that individuals choose to join the intervention group or the control group voluntarily based on personal characteristics such as motivation and needs. This will lead to between-group differences, making it impossible to accurately attribute the intervention outcomes to the intervention itself. In this study, participants were assigned using the block randomization method. First, all participants were ranked in descending order according to their scores on the self-injury scale. Participants with similar scores were paired up, and each pair was then randomly allocated to the experimental group and the control group respectively. This grouping approach can minimize the confounding influence of the self-selection effect.

The regression effect refers to a tendency that when individuals obtain extremely high or low scores on measurements such as depression and anxiety scales, their subsequent scores will generally regress toward the group mean rather than remaining at extreme levels, even in the absence of targeted interventions. The natural recovery effect refers to the phenomenon in which an individual’s psychological problems diminish or disappear on their own over time without receiving any specific treatment or professional intervention. In this study, a control group was included. Participants in the control group did not show improvements comparable to those in the intervention group over the same period, which to a certain extent rules out regression to the mean or natural recovery effect as the primary explanation for the observed changes.

In terms of baseline differences, there were no significant differences between the intervention group and the control group in levels of depression and self-injurious behavior. This result indicates that the two groups were well homogeneous prior to the intervention, which effectively eliminates the confounding effects of baseline disparities on the evaluation of intervention outcomes.

The social desirability effect in psychological intervention refers to the tendency of participants to give overly positive feedback to interventionists immediately after the intervention. This inclination stems partly from a sense of gratitude towards the interveners and partly from a desire to justify the value of their time investment, which may unintentionally exaggerate the immediate effects of the intervention. To avoid such bias, a two-month follow-up assessment was carried out. Participants’ anxiety, depression and self-injury levels kept declining or stayed steady. It can thus be concluded that the immediate post-intervention evaluation was relatively less affected by the social desirability effect, and the intervention effects were well sustained.

In addition, four co-facilitating students were involved throughout the intervention. These students actively engaged in the intervention activities and voluntarily shared their personal feelings and experiences. Their high level of involvement motivated other group members to interact, enabling them to serve as positive peer role models, and effectively boosted disclosure among members and group dynamics within the group. However, according to the contrast effect in social comparison theory, such peer models may trigger peer pressure and social desirability effects, leading participants to avoid expressing their genuine negative emotions. In the present intervention, the ratio of peer facilitators to participants was 1:7. Given the small proportion of facilitators, no prominent peer pressure or social desirability effects emerged.

### 4.2 Highlights

Compared with previous self-injury interventions, this study has the following highlights. First, regarding intervention methodology, this study differs from prior research that directly adopts established therapies. Instead, it draws on the Process Model of Emotion Regulation and focuses on five core adaptive strategies: distraction, emotional expression, self-focused cognitive change, situation-focused cognitive change, and problem-solving. These strategies were systematically incorporated into a complete intervention program, which enhanced emotion regulation among individuals with self-injurious behaviors.

Second, this study addressed prevalent real-life challenges among college students — including interpersonal difficulties, academic stress and career confusion — and developed targeted intervention activities, such as family drawing, interpersonal psychodrama, sharing of learning experiences and strategies, the “Past • Present • Future” drawing exercise, and goal-setting planning. These customized approaches improve the practicality and efficacy of the intervention.

Third, in the evaluation of intervention effects, this study adopted open-ended surveys in addition to self-report scales. Qualitative data were coded and analyzed using the NVivo software. An evaluation system for self-injury interventions was developed, comprising three first-level and nine second-level indicators. This integrated method comprehensively and thoroughly captures the various benefits brought by the intervention.

### 4.3 Practical implications

This study holds significant implications for self-injury intervention. It is crucial to assist self-injurious individuals in adopting multiple adaptive emotion regulation strategies and enhancing their emotional regulation capacity. To reduce self-injurious behaviors, interventions can be designed based on the Process Model of Emotion Regulation, incorporating strategies such as distraction, emotional expression, self-focused cognitive reappraisal, situation-focused cognitive reappraisal, and problem-solving. These can be implemented through various psychological techniques including relaxation training, music appreciation, drawing tests, and psychodrama. Furthermore, it is essential to address the diverse stressors faced by different individuals engaging in self-injury and to develop tailored intervention measures accordingly. Clinically, apart from pharmacotherapy (52), the group counseling protocol developed in the present study may be utilized to alleviate non-suicidal self-injury behaviors.

### 4.4 Limitations and future directions

This study has several limitations. First, the sample size in this study, while meeting minimum requirements, was limited. Future studies should recruit a larger sample and apply Growth Mixture Modeling (GMM) to better capture the heterogeneity in intervention effects by analyzing differential change trajectories across participant subgroups.

Second, the intervention group and control group in this study were not fully matched. Although there were no significant differences between the two groups in the levels of depression and self-injurious behaviors, the intervention group had significantly a higher level of anxiety than the control group. Furthermore, the study did not measure or match the two groups on self-change motivation. Future studies should more rigorously control for between-group differences to further validate the effectiveness of this intervention.

Third, regarding the follow-up duration, this study only tracked participants for a period of two months. Such a short observation period may not be sufficient to fully capture the long-term effects of the intervention. Therefore, future research should extend the follow-up period (e.g., to six months or one year) to more rigorously examine the long-term efficacy of this intervention protocol.

Fourth, only self-report measures were adopted to evaluate the effects of the intervention in this study. This approach is prone to inherent biases specific to self-assessment, such as social desirability, self-concealment, and response sets. Future research may integrate rater-rated assessments and behavioral observations to implement multi-source measurement. Triangulation of multi-faceted data can enhance the ecological validity of measurements and address the absence of blinded outcome assessment.

Fifth, participants in the control group of this study only received routine campus mental health popularization education, with relatively simple treatment arrangements. As a result, therapist attention, expectancy effects and the influence of group support could not be well controlled. Future studies may deliver standardized general mental health education exclusively to the control group to better equalize the experimental conditions between the intervention and control groups.

Sixth, this study used a single-item measure to assess intervention satisfaction, which provides limited evidence for its reliability and validity. Future studies could develop a multi-item scale to improve the reliability and validity of intervention satisfaction measurement.

Seventh, this study did not conduct systematic assessment or management of self-injurers’ previous suicide attempts, current suicidal ideation, severe depression, psychosis, acute crisis, and other relevant clinical risk conditions. Future interventions may strengthen the assessment and management of these aspects.

Eighth, the intervention participants in this study were limited to college students with self-injurious behaviors. It is important to note that self-injurious behavior generally results from the accumulation of multiple risk factors (53), and the combination of risk factors varies across individuals. Taking postpartum women as an example, typical risk factors may include physical recovery, newborn care, financial stress, and work-family conflict (54). Therefore, intervention programs should be tailored to the specific characteristics of different self- injurious groups. Building on the Process Model of Emotion Regulation, future research could incorporate the risk characteristics of different populations to design contextually adaptable and specific intervention programs, thereby expanding the applicability and effectiveness of this intervention approach on the existing basis.

## 5 Conclusions

Drawing on the Process Model of Emotion Regulation, this study developed a preliminary group intervention integrating multiple emotion regulation strategies (distraction, emotional expression, cognitive restructuring, and problem-solving). The program appears feasible and acceptable, and may alleviate anxiety, depression, and non-suicidal self-injury (NSSI) behaviors in college students.

## Data Availability

The original contributions presented in the study are included in the article/supplementary material. Further inquiries can be directed to the corresponding author.
